# Uncovering Promising Practices for Supporting Older People Experiencing Homelessness

**DOI:** 10.1093/geroni/igaa057.2489

**Published:** 2020-12-16

**Authors:** Sarah Canham, Joe Humphries, Victoria Burns, Tamara Sussman, Christine Walsh

**Affiliations:** 1 University of Utah, Salt Lake City, Utah, United States; 2 Simon Fraser University, Vancouver, British Columbia, Canada; 3 University of Calgary, Calgary, Alberta, Canada; 4 McGill University, Montreal, Ontario, Canada

## Abstract

Montréal, Calgary, and Vancouver have seen a dramatic increase in homelessness among adults aged 50+. In order to identify ‘promising practices’ that promote aging-in-the-right-place for older people experiencing homelessness (OPEH) in Montréal, Calgary, and Vancouver, we conducted an environmental scan and three World Café workshops with 99 service providers and OPEH. We identified 53 promising practices managed or operated by 42 providers which we categorized across a shelter/housing continuum: 1) Emergency/transitional/temporary shelter/housing; 2) Independent housing with offsite supports; 3) Supported independent housing with onsite, non-medical supports; 4) Permanent supportive housing with onsite medical support and/or specialized services; 5) Long-term care; and 6) Palliative care/hospice. Study findings provide a template for existing solutions to the diverse shelter/housing needs of OPEH and insight into the gaps in shelter/housing and services that would support OPEH to age-in-the-right place. Policy and practice implications for scaling promising practices will be discussed. Part of a symposium sponsored by the Environmental Gerontology Interest Group.

